# Conceptus-derived cytokines interleukin-1β and interferon-γ induce the expression of acute phase protein serum amyloid A3 in endometrial epithelia at the time of conceptus implantation in pigs

**DOI:** 10.5713/ab.22.0334

**Published:** 2022-11-14

**Authors:** Soohyung Lee, Inkyun Yoo, Yugyeong Cheon, Hakhyun Ka

**Affiliations:** 1 Division of Biological Science and Technology, Yonsei University, Wonju, 26493, Korea

**Keywords:** Cytokine, Endometrium, Pig, Pregnancy, Serum Amyloid A3, Somatic Cell Nuclear Transfer

## Abstract

**Objective:**

Serum amyloid A3 (SAA3), an acute phase response protein, plays important roles in opsonization, antimicrobial activity, chemotactic activity, and immunomodulation, but its expression, regulation, and function at the maternal–conceptus interface in pigs are not fully understood. Therefore, we determined the expression of *SAA3* in the endometrium throughout the estrous cycle and at the maternal–conceptus interface during pregnancy.

**Methods:**

Endometrial tissues from pigs at various stages of the estrous cycle and pregnancy and with conceptuses derived from somatic cell nuclear transfer (SCNT), conceptus tissues during early pregnancy, and chorioallantoic tissues during mid- to late pregnancy were obtained and the expression of *SAA3* was analyzed. The effects of the steroid hormones, interleukin-1β (IL1B), and interferon-γ (IFNG) on the expression of *SAA3* were determined in endometrial explant cultures.

**Results:**

*SAA3* was expressed in the endometrium during the estrous cycle and pregnancy, with the highest level on day 12 of pregnancy. The expression of *SAA3* in the endometrium was significantly higher on day 12 of pregnancy than during the estrous cycle. Early-stage conceptuses and chorioallantoic tissues during mid to late pregnancy also expressed *SAA3*. The expression of *SAA3* was primarily localized to luminal epithelial cells in the endometrium. In endometrial explant cultures, the expression of *SAA3* was induced by increasing doses of IL1B and IFNG. Furthermore, the expression of *SAA3* decreased significantly in the endometria of pigs carrying conceptuses derived from SCNT on day 12 of pregnancy.

**Conclusion:**

These results suggest that the expression of *SAA3* in the endometrium during the implantation period increases in response to conceptus-derived IL1B and IFNG. The failure of those appropriate interactions between the implanting conceptus and the endometrium leads to dysregulation of endometrial *SAA3* expression, which could result in pregnancy failure. In addition, SAA3 could be a specific endometrial epithelial marker for conceptus implantation in pigs.

## INTRODUCTION

Appropriate interactions between the maternal endometrium and the implanting conceptus are essential for the establishment and maintenance of pregnancy in mammals [1]. In pigs, conceptuses implanting in the uterine lumen between days 10 and 12 of pregnancy dramatically change their morphological shape, elongating from spherical to filamentous form to increase the surface area for attachment and to initiate noninvasive implantation into the endometrium [2]. During this period, conceptuses secrete a variety of signaling molecules, including estrogen, interleukin-1β2 (IL1B2), and prostaglandins (PGs), into the uterine lumen. Then, between days 13 and 18 of pregnancy, the conceptuses produce interferons (IFNs), particularly IFN-γ (IFNG) and IFN-δ [3]. In response to those conceptus signals, the endometrium undergoes morphological and functional changes to induce adhesion of the conceptus trophectoderm and support growth and differentiation of the implanting conceptus [2]. Specifically, the endometrium increases the expression of a variety of genes involved in conceptus attachment, immunity, and PG synthesis and transport [1–3]. Although many endometrial factors induced by conceptus signals at the time of conceptus implantation have been studied, the cellular and molecular functions of those factors at the maternal–conceptus interface in regulating the establishment and maintenance of pregnancy are not completely understood.

Previously, we identified differentially expressed genes (DEGs) in the endometrium between day 12 of the estrous cycle and pregnancy in search of factors involved in the maternal–conceptus interaction during conceptus implantation in pigs. Those DEGs include inhibitor of DNA binding 2, S100 calcium binding protein A7A (*S100A7A*), salivary lipocalin 1, serum amyloid A3 (*SAA3*), and transient receptor potential vanilloid type 6 [4]. Among them, the SAA family proteins, including SAA3, are well-known biomarkers for a variety of inflammatory diseases [5–7]. The functions of SAAs include opsonization and elimination of invading pathogens, recruitment of immune cells to inflammatory sites, metabolism and transport of cholesterol, degradation of extracellular matrix, development of many acute and chronic inflammatory diseases, and cancer metastasis [5–7]. In pigs, the *SAA* family is composed of four genes, *SAA1*, *SAA2*, *SAA3*, and *SAA4*, of which *SAA1* is considered a pseudogene. *SAA2*, *SAA3*, and *SAA4* are differentially induced during an acute phase response [8]. The expression of *SAA2* and *SAA4* is induced mainly in the liver, whereas the expression of *SAA3*, the major circulating isoform in pigs, is induced in various tissues, including the liver, lungs, and spleen [8,9]. The expression of the SAA family is induced by inflammatory cytokines, including tumor necrosis factor-α (TNF-α), IL1, IL6, and IFNG, along with vitamin A in humans and mice [10]. The expression of SAAs increases in the endometrium with endometritis in cattle and mares [11] and in endometrial carcinoma in humans [12]. SAA proteins affect trophoblast invasion by inducing the expression of matrix metalloproteinase 2 in humans [13]. Although the expression and function of the SAA family in the endometrium have been studied in humans and some domestic animals, the expression, function, and regulation of the SAA family in the endometrium during pregnancy in pigs have not been fully studied.

The somatic cell nuclear transfer (SCNT) technique has a broad range of potential applications, including rescue of endangered species, production of transgenic animals, cell transplantation, disease modeling, and regenerative medicine [14]. However, the SCNT technique has extremely low cloning efficiency and is associated with abnormalities of cloned animals, mainly due to incomplete reprogramming of the donor cell nucleus in SCNT-cloned embryos and inappropriate maternal–conceptus interactions in the uterus during pregnancy [15]. Some studies of SCNT-cloned embryos in pigs have shown altered expression of many endometrial and placental genes at the maternal–conceptus interface [16–19]. However, no one has yet determined whether SAAs are appropriately expressed in an endometrium with SCNT-derived embryos and fully explored the mechanisms of those maternal–conceptus interactions in the endometrium with SCNT-cloned embryos.

Therefore, to examine the role of the SAA family during pregnancy in pigs, we determined i) expression of *SAA3*, the major isoform induced in various tissues in pigs, in the endometrium throughout the estrous cycle and pregnancy; ii) localization of *SAA3* mRNA at the maternal–conceptus interface; iii) expression of *SAA3* in conceptus tissues during early pregnancy and in chorioallantoic tissues during mid to term pregnancy; iv) regulation of *SAA3* expression by steroid hormones and IL1B and IFNG in endometrial tissues; and v) expression of *SAA3* in the endometria of gilts with SCNT-derived conceptuses on day 12 of pregnancy.

## MATERIALS AND METHODS

### Animals and tissue preparation

All experimental procedures involving animals were conducted in accordance with the Guide for the Care and Use of Research Animals in Teaching and Research and approved by the Institutional Animal Care and Use Committee of Yonsei University (No. YWC-P120) and the National Institute of Animal Science (No. 2015-137). Sexually mature, crossbred female gilts of similar age (6 to 8 months) and weight (100 to 120 kg) were assigned randomly to either cyclic or pregnant status. Gilts assigned to the pregnant uterus status group were artificially inseminated with fresh boar semen at the onset of estrus (day 0) and 12 h later. The reproductive tracts of the gilts were obtained immediately after slaughter on day 0, 3, 6, 9, 12, 15, or 18 of the estrous cycle (21 days of cycle; days 0–3, estrus; days 3–6, metestrus; days 6–15, diestrus; days 15–0, proestrus) or on day 10, 12, 15, 30, 60, 90, or 114 of pregnancy (n = 3–6 gilts/d/status). Pregnancy was confirmed by the presence of apparently normal spherical to filamentous conceptuses in uterine flushings on days 12 and 15 and the presence of embryos and placenta on subsequent days. Uterine flushings were obtained by introducing and recovering 50 mL of phosphate buffered saline (PBS, pH 7.4) at tissue collection (25 mL/uterine horn). Chorioallantoic tissues were obtained from days 30, 60, 90, and 114 of pregnancy (n = 3–4 gilts/d). Endometrial tissues were also obtained from four gilts carrying embryos generated by SCNT on day 12 of pregnancy, as described previously [16,17]. Some ovoidal, tubular, and elongating conceptuses were recovered in uterine flushings from the uteri of pigs with SCNT-derived embryos. Endometrial tissues dissected free of myometrium and collected from the middle portion of each uterine horn, conceptus tissues obtained in uterine flushings and washed with PBS (pH 7.4), and chorioallantoic tissues were snap-frozen in liquid nitrogen and stored at −80°C prior to RNA extraction. For *in situ* hybridization, cross-sections of endometrium were fixed in 4% paraformaldehyde in PBS (pH 7.4) for 24 h and then embedded in paraffin, as previously described [20].

### Endometrial explant cultures

To determine the effects of estradiol-17β (E_2_), progesterone (P_4_), and IL1B on the expression of *SAA3* mRNA in the endometrium, endometrial explant tissues obtained from gilts on day 12 of the estrous cycle were cultured as previously described [20]. Endometrium dissected from the myometrium was placed into warm phenol red–free Dulbecco’s modified Eagle’s medium/F-12 culture medium (DMEM/F-12; Sigma, St. Louis, MO, USA) containing penicillin G (100 IU/mL) and streptomycin (0.1 mg/mL). The endometrium was minced with scalpel blades into small pieces (2 to 3 mm^3^), and aliquots of 500 mg were placed into T25 flasks with serum-free modified DMEM/F-12 containing 10 μg/mL insulin (Sigma, USA), 10 ng/mL transferrin (Sigma, USA), and 10 ng/mL hydrocortisone (Sigma, USA). Immediately after mincing, the endometrial explants were cultured in the presence of ethanol (control), E_2_ (10 ng/mL; Sigma, USA), P_4_ (30 ng/mL; Sigma, USA), P_4_+E_2_, P_4_+E_2_+ICI182,780 (ICI; an estrogen receptor antagonist; 200 ng/mL; Tocris Bioscience, Ellisville, MO, USA), or P_4_+E_2_+RU486 (RU; a progesterone receptor antagonist; 30 ng/mL; Sigma, USA) for 24 h with rocking in an atmosphere of 5% CO_2_ in air at 37°C. To determine the effect of IL1B on the expression of endometrial *SAA3*, explant tissues were treated with 0, 1, 10, or 100 ng/mL IL1B (Sigma, USA) in the presence of both E_2_ (10 ng/mL) and P_4_ (30 ng/mL) for 24 h at 37°C. To determine the effect of IFNG on the expression of endometrial *SAA3*, endometrial explants were cultured with rocking in the presence of E_2_ (10 ng/mL), P_4_ (30 ng/mL), and IL1B (10 ng/mL) for 24 h in an atmosphere of 5% CO_2_ in air at 37°C and then for an additional 24 h with 0, 1, 10, or 100 ng/mL of IFNG (R&D Systems, Minneapolis, MN, USA) in the presence of E_2_ (10 ng/mL), P_4_ (30 ng/mL), and IL1B (10 ng/mL), as previously described [21]. Explant tissues were then harvested, and total RNA was extracted for real-time reverse transcription–polymerase chain reaction (RT-PCR) to determine the expression level of *SAA3* mRNA. These experiments were conducted using endometria from three gilts on day 12 of the estrous cycle, and treatments were performed in triplicate using tissues obtained from each of the three gilts.

### Total RNA extraction, RT-PCR, and cloning of porcine *SAA3* cDNA

Total RNA was extracted from endometrial, conceptus, and chorioallantoic tissues using TRIzol reagent (Invitrogen, Carlsbad, CA, USA) according to the manufacturer’s recommendations. The quantity of RNA was assessed spectrophotometrically, and the integrity of RNA was validated following electrophoresis in 1% agarose gel. Four micrograms of total RNA were treated with DNase I (Promega, Madison, WI, USA) and reverse transcribed using SuperScript II Reverse Transcriptase (Invitrogen, USA) to obtain cDNA. The cDNA templates were then diluted 1:4 with sterile water and amplified by PCR using Taq polymerase (Takara Bio, Shiga, Japan). The final PCR reaction volume of 50 μL included 3 μL of cDNA, 5 μL of 10× PCR buffer, 4 μL of dNTP mix (2.5 mM), 1 μL of each primer (20 μM), 0.3 μL of Taq polymerase (Takara Bio, Japan), and 36.7 μL of H_2_O. Initial denaturation was performed at 94°C for 5 min; 40 cycles of amplification were carried out at 94°C for 30 s, 60°C for 30 s, and 72°C for 30 s; and final extension was conducted at 72°C for 10 min. The PCR products were separated on 2% agarose gels and visualized by ethidium bromide staining. The identity of each amplified PCR product was verified by sequence analysis after being cloned into the pCRII vector (Invitrogen, USA).

### Quantitative real-time reverse transcription–polymerase chain reaction

The level of expression of *SAA3* cDNA in endometrial and chorioallantoic tissues was analyzed by real-time RT-PCR using an Applied Biosystems StepOnePlus system (Applied Biosystems, Foster City, CA, USA) and the SYBR Green method. Power SYBR Green PCR Master Mix (Applied Biosystems, USA) was used for the PCR reactions. The final reaction volume of 20 μL included 2 μL of cDNA, 10 μL of 2X Master mix, 2 μL of each primer (2 μM), and 4 μL of ddH_2_O. PCR was performed with an initial incubation at 95°C for 10 min, followed by 40 cycles of 15 s at 95°C and 30 s at 60°C. The sequences of the primer pairs are listed in Table 1. The results are reported as expression relative to that detected on day 12 of the estrous cycle or that detected in control explant tissues after normalization of the transcript amount to the geometric mean of the endogenous ribosomal protein L7 (*RPL7*), ubiquitin B (*UBB*), and TATA binding protein (*TBP*) controls by the 2^–ΔΔCT^ method, as previously described [22].

### Nonradioactive *in situ* hybridization

Nonradioactive *in situ* hybridization was performed to determine the localization of *SAA3* expression in the uterine endometrium, as previously described with some modifications [23]. Sections (5 μm thick) were rehydrated through successive baths of xylene, 100% ethanol, 95% ethanol, diethylpyrocarbonate (DEPC)-treated water, and DEPC-treated PBS. Tissue sections were boiled in citrate buffer (pH 6.0) for 10 min. After being washed in DEPC-treated PBS, they were digested using 5 μg/mL Proteinase K (Sigma, USA) in TE (100 mM Tris-HCl, 50 mM ethylenediaminetetraacetic acid, pH 7.5) at 37°C. After post-fixation in 4% paraformaldehyde, tissue sections were incubated twice for 15 min each in PBS containing 0.1% active DEPC and then equilibrated for 15 min in 5× saline sodium citrate (SSC) buffer. The sections were prehybridized for 2 h at 68°C in a hybridization mix (50% formamide, 5× SSC, 500 μg/mL herring sperm DNA, 250 μg/mL yeast tRNA). Sense and antisense riboprobes for each gene were generated using partial cDNAs cloned into pCRII vectors by linearization with appropriate restriction enzymes, and they were labeled with digoxigenin (DIG)-UTP using a DIG RNA labeling kit (Roche, Indianapolis, IN, USA). The probes were denatured for 5 min at 80°C and added to the hybridization mix. The hybridization reaction was carried out overnight at 68°C. Prehybridization and hybridization reactions were performed in a box saturated with a 5× SSC 50% formamide solution to prevent evaporation, and no coverslips were used. After hybridization, sections were washed for 30 min in 2× SSC at room temperature, 1 h in 2× SSC at 65°C, and 1 h in 0.1× SSC at 65°C. Probes bound to the section were detected immunologically using sheep anti-DIG Fab fragments covalently coupled to alkaline phosphatase and nitro blue tetrazolium chloride/5-bromo-4-chloro-3-indolyl phosphate (toluidine salt) as a chromogenic substrate, according to the manufacturer’s protocol (Roche, USA).

### Statistical analysis

Data from real-time RT-PCR for *SAA3* expression were subjected to analysis of variance (ANOVA) using the general linear model procedures of SAS (Cary, NC, USA). As sources of variation, the model included day, pregnancy status (cyclic or pregnant, days 12 and 15 post-estrus), and their interactions to evaluate the steady-state level of *SAA3* mRNA. Data from real-time RT-PCR were analyzed by least squares regression analysis to assess the effects of days of the estrous cycle (days 0, 3, 6, 9, 12, 15, and 18) and of pregnancy (days 10, 12, 15, 30, 60, 90, and 114) in endometrial tissues, the effects of day of pregnancy (days 30, 60, 90, and 114) in chorioallantoic tissues, and the effects of IL1B and IFNG doses in explant tissues on the expression of *SAA3*. Preplanned orthogonal contrasts (control vs E_2_; control vs P_4_; P_4_ vs P_4_+E_2_; P_4_+E_2_ vs P_4_+E_2_+ICI; and P_4_+E_2_ vs P_4_+E_2_+RU) were used to test the effects of hormone treatments, and one-way ANOVA followed by the Tukey post-test was used to evaluate the effects of IL1B and IFNG on endometrial *SAA3* expression in the explant cultures. Data from real-time RT-PCR analysis for comparison of *SAA3* mRNA levels in the endometrium with SCNT and non-SCNT conceptuses were subjected to the Student’s T-test. Prior to the analyses, all data were tested for normality and homogeneity of variance, and log transformation was performed when necessary. Data are presented as mean with standard error of the mean. p-values less than 0.05 were considered significant, and p-values of 0.05 to 0.10 were considered to indicate a trend toward significance.

## RESULTS

### Expression of *SAA3* in the endometrium during the estrous cycle and pregnancy

**Figure 1:**
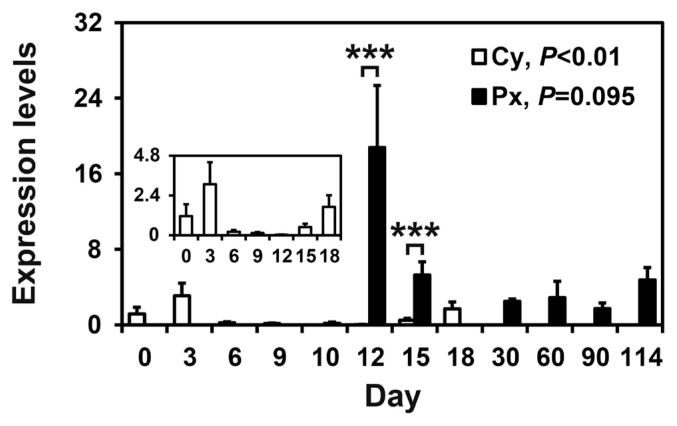
Expression of serum amyloid A3 (*SAA3*) in the endometrium during the estrous cycle and pregnancy. Endometrial tissue samples from cyclic (Cy) and pregnant gilts (Px) were analyzed by real-time reverse transcription-polymerase chain reaction, and data are reported as expression relative to that detected on day 0 of the estrous cycle after normalization of the transcript amount to the endogenous *RPL7*, *UBB*, and *TBP* controls. *RPL7*, ribosomal protein L7; *UBB*, ubiquitin B; *TBP*, TATA binding protein. Data are presented as mean with standard error. *** p<0.001.

To determine whether the expression of *SAA3* mRNA in the endometrium changed during the estrous cycle and pregnancy in pigs, we examined the relative abundance of *SAA3* mRNA in the endometrium using real-time RT-PCR (Figure 1). The expression of *SAA3* in the endometrium changed stage-specifically during the estrous cycle (quadratic effect of day, p<0.01) and tended to change during pregnancy, with the highest level on day 12 of pregnancy (linear effect of day, p = 0.095). On days 12 and 15 post-estrus, *SAA3* expression was affected by pregnancy status (p<0.001), and the expression of *SAA3* was greater on day 12 and day 15 of pregnancy than on day 12 (p<0.001) and day 15 (p<0.001) of the estrous cycle, respectively.

### Expression of *SAA3* in conceptuses on days 12 and 15 of pregnancy and chorioallantoic tissues during the later stages of pregnancy

**Figure 2:**
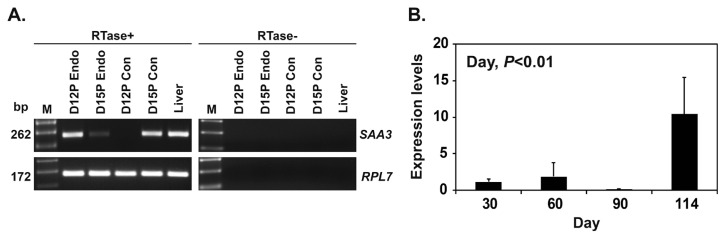
Expression of serum amyloid A3 (*SAA3*) in conceptuses from days 12 and 15 of pregnancy and in chorioallantoic tissues during mid to late pregnancy. (A) RT-PCR analysis of *SAA3* mRNAs in conceptuses on days 12 and 15 of pregnancy. *RPL7* was used as a positive control for the PCR reaction, and liver tissue was used as a positive control. RTase +/-, with (+) or without (-) reverse transcriptase; M, molecular marker; D12 Endo, endometrium on day 12 of pregnancy; D12 Con, day 12 conceptus; D15 Con, day 15 conceptus. (B) Real-time RT-PCR analysis of the expression of *SAA3* in chorioallantoic tissues on days 30, 60, 90, and 114 of pregnancy. Data are reported as expression relative to that detected on day 30 of pregnancy after normalization of the transcript amount to the endogenous *RPL7*, *UBB*, and *TBP* controls, and data are presented as mean with standard error. RT-PCR, reverse transcription-polymerase chain reaction; *RPL7*, ribosomal protein L7; *UBB*, ubiquitin B; *TBP*, TATA binding protein.

To determine whether conceptuses expressed *SAA3* mRNA during early pregnancy, we performed RT-PCR using cDNA from conceptuses on days 12 and 15 of pregnancy. We found that *SAA3* mRNA was expressed in conceptuses on day 15 of pregnancy, as well as in the liver that was used as a positive control tissue (Figure 2A). Real-time RT-PCR analysis showed that the expression of *SAA3* in chorioallantoic tissues during mid to term pregnancy (days 30 to 114) increased (quadratic effect of day, p<0.01) (Figure 2B).

### Localization of *SAA3* mRNA in the endometrium on days 12 and 15 of the estrous cycle and pregnancy

**Figure 3:**
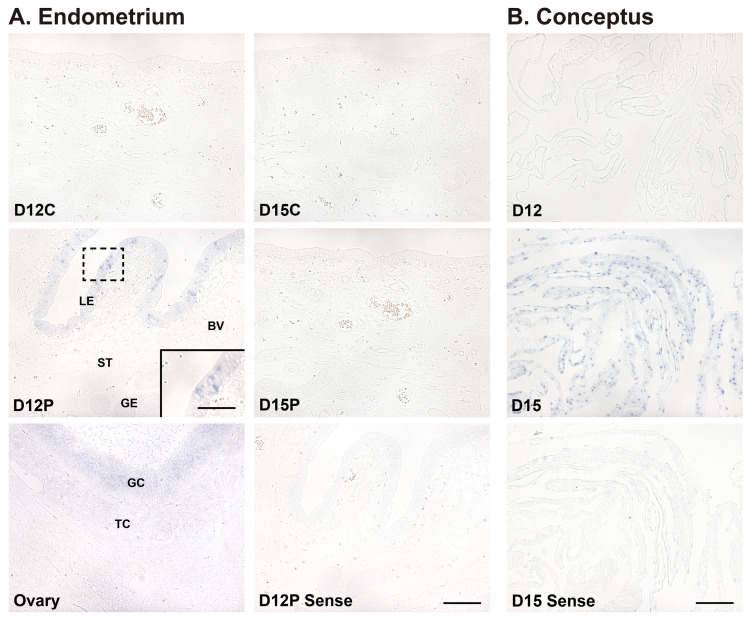
*In situ* hybridization analysis of serum amyloid A3 (*SAA3*) expression in the endometrium (A) and conceptus tissues (B). (A) Representative images on days 12 and 15 of the estrous cycle and pregnancy are shown. A uterine section from day 12 of pregnancy hybridized with DIG-labeled sense *SAA3* cDNA probe (Sense) is shown as the negative control, and a tissue section from the ovary served as a positive control. D, day; C, estrous cycle; P, pregnancy; LE, luminal epithelium; GE, glandular epithelium; ST, stroma; BV, blood vessel, GC, granulosa cell; TC, theca cell. Bars = 100 μm and 20 μm in inset. (B) *In situ* hybridization analysis of *SAA3* mRNA in conceptus tissues on days 12 and 15 of pregnancy. A conceptus tissue section from day 15 of pregnancy hybridized with DIG-labeled sense *SAA3* cDNA probe (Sense) was the negative control. D, day; P, pregnancy. Bar = 100 μm.

Localization of *SAA3* mRNA was determined by *in situ* hybridization in the endometrium on days 12 and 15 of the estrous cycle and pregnancy and in conceptuses on days 12 and 15 of pregnancy in pigs (Figure 3). The expression of *SAA3* mRNA was localized predominantly to luminal epithelial (LE) cells in the endometrium, with strong signal intensity on day 12 of pregnancy. *SAA3* mRNA was also detected in the ovarian tissue that was used as a positive control [24] (Figure 3A). *SAA3* mRNA was barely detectable in conceptus tissues on day 12 of pregnancy and was readily detectable in trophectoderm and endoderm cells on day 15 of pregnancy (Figure 3B).

### Effects of steroid hormones, E_2_ and P_4_, and cytokines IL1B and IFNG on *SAA3* mRNA expression in endometrial tissues

**Figure 4:**
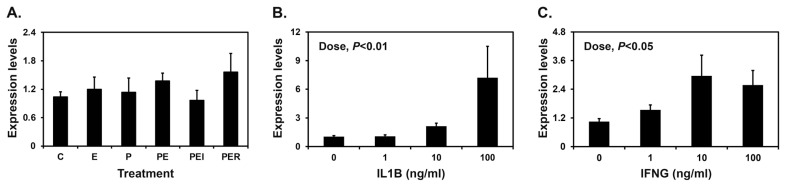
Effects of steroid hormones (A), IL1B (B), and IFNG (C) on the expression of serum amyloid A3 (*SAA3*) in endometrial explant cultures. Endometrial explants from gilts on day 12 of the estrous cycle were cultured (A) with steroid hormones [control (C), E_2_ (E), P_4_ (P), E_2_+P_4_ (PE), E_2_+P_4_+ICI (ICI, an estrogen receptor antagonist) (PEI), or E_2_+P_4_+RU (RU; a progesterone receptor antagonist) (PER)], (B) with increasing doses of IL1B (0, 1, 10, and 100 ng/mL) in the presence of E_2_ (estradiol-17*β*; 10 ng/mL) and P_4_ (progesterone; 30 ng/mL), or (C) with increasing doses of IFNG (0, 1, 10, and 100 ng/mL) in the presence of E_2_ (10 ng/mL) and P_4_ (30 ng/mL). The abundance of mRNA expression in the real-time RT-PCR analyses is relative to that for *SAA3* mRNA in the control group of endometrial explants after normalization of the transcript amounts to *RPL7*, *UBB*, and *TBP* mRNAs. Data are presented as mean with standard error. For each treatment, experiments were performed with endometria from eight gilts. These treatments were performed in triplicate using tissues obtained from each of three gilts. RT-PCR, reverse transcription-polymerase chain reaction; *RPL7*, ribosomal protein L7; *UBB*, ubiquitin B; *TBP*, TATA binding protein.

Implanting conceptuses secrete E_2_ and IL1B into the uterine lumen, with the greatest abundance on day 12 of pregnancy, along with IFNG on day 15 of pregnancy. Furthermore, P_4_ from the corpus luteum regulates the expression of many endometrial genes in pigs [1]. Therefore, we determined the effects of E_2_, P_4_, IL1B, and IFNG on the expression of *SAA3* mRNA in endometrial explant tissues. Endometrial *SAA3* mRNA levels were not changed by E_2_ or P_4_ (Figure 4A). However, treatment with IL1B and IFNG increased the expression of *SAA3* in a dose-dependent manner in endometrial tissues (linear effect of dose for IL1B, p<0.01; linear effect of dose for IFNG, p<0.05) (Figure 4B and 4C).

### Expression and localization of *SAA3* mRNA in endometria carrying conceptuses derived from SCNT or natural mating

**Figure 5:**
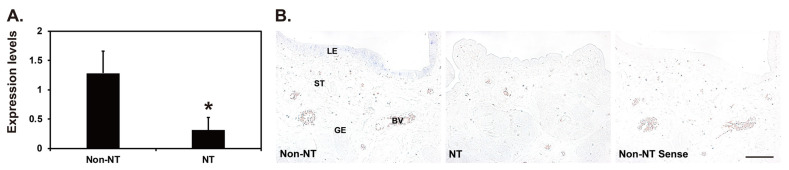
Expression of serum amyloid A3 (*SAA3*) mRNA in the endometria of gilts carrying conceptuses derived from natural mating (Non-NT) or somatic cell nuclear transfer-cloned conceptuses (NT) on day 12 of pregnancy. (A) Real-time reverse transcription-polymerase chain reaction analysis of *SAA3* mRNA in endometrial tissues from Non-NT and NT pregnancies. Data are reported as expression relative to that detected on day 12 of a Non-NT pregnancy after normalization of the transcript amount to the endogenous ribosomal protein L7 control. Data are presented as mean with standard error. * p<0.05. (B). *In situ* hybridization analysis of *SAA3* mRNA in endometrial tissues from Non-NT and NT pregnancies. A representative uterine section from a Non-NT pig on day 12 of pregnancy hybridized with a digoxigenin-labeled sense *SAA3* cRNA probe (Sense) is shown as a negative control for *in situ* hybridization. LE, luminal epithelia; GE, glandular epithelia; St, stroma; BV, blood vessel. Bar = 100 μm.

Our real-time RT-PCR analysis showed that the expression of *SAA3* mRNA in the endometrial tissues of gilts carrying conceptuses derived from SCNT was lower than that in gilts carrying conceptuses from natural mating on day 12 of pregnancy (p<0.05) (Figure 5A). *In situ* hybridization analysis showed that *SAA3* expression was readily detectable in the LE cells of endometria with conceptuses from natural mating, whereas *SAA3* expression was rarely detected in the LE cells of endometria with conceptuses derived from SCNT (Figure 5B).

## DISCUSSION

The novel findings of this study in pigs are: i) *SAA3* is expressed in the endometrium during the estrous cycle and pregnancy in a status- and stage-specific manner; ii) conceptus tissues during early pregnancy and chorioallantoic tissues from day 30 to term pregnancy express *SAA3*; iii) *SAA3* expression is primarily in the endometrial LE on day 12 of pregnancy and in conceptuses on day 15 of pregnancy; iv) IL1B and IFNG induce the expression of *SAA3* in endometrial explant tissues; and v) the expression of *SAA3* in the endometria of gilts with SCNT-derived conceptuses was lower than that in gilts with conceptuses from natural mating on day 12 of pregnancy.

SAAs are acute phase proteins, which means that the levels in plasma increase rapidly in response to acute phase responses such as inflammation, infection, and trauma [10]. Because excessive SAA levels lead to SAA deposition, which causes amyloidosis under recurrent or chronic inflammatory conditions, SAA protein is used as a blood biomarker to evaluate and monitor disease severity in patients with amyloidosis or inflammatory rheumatic disease [10]. Several studies also suggest that SAA is associated with pregnancy complications such as infections, endometrial cancer, and preeclampsia. In humans, blood level of SAA does not change during normal pregnancy, but does increase in pregnancies affected by infection or preeclampsia and in patients with endometrial endometrioid carcinoma [25]. In goats, *SAA3* mRNA is expressed in the uterus, blood SAA protein level increases during the periparturient period of pregnancy, and exclusive SAA3 deposition in uterine caruncular stroma causes amyloidosis, leading to fetal death [26,27]. Our results in this study clearly show that *SAA3* is expressed in the endometrium at increased level during the implantation period in pigs. Although SAAs are valuable biomarkers for pregnancy complications such as infections, preeclampsia, and endometrioid carcinoma in some species, *SAA3* is expressed in the uterus during normal pregnancy in pigs. It is thought that the maternal–conceptus interface is mainly regulated by a pro-inflammatory condition during the implantation period [28]. Thus, the increased *SAA3* expression we observed reflects the pro-inflammatory condition between the endometrium and the conceptus during this period in pigs. In addition, *SAA3* could be a good marker for conceptus implantation in pigs because *SAA3* expression is uniquely increased at that time.

SAA production is induced by inflammatory cytokines such as IL1B, IL6, IFNG, and TNF-α and microbial components such as lipopolysaccharide in various cell types [10]. Because the expression of *SAA3* in the endometrium was greatest on day 12 during pregnancy and implanting porcine conceptuses secrete estrogen and cytokines, IL1B and IFNG, we postulated that the conceptus-derived estrogen and cytokines would increase endometrial *SAA3* expression. Estrogen of conceptus origin is responsible for inducing the expression of many endometrial genes, such as aldo-keto reductase 1B1, fibroblast growth factor 7, IFN-alpha and beta receptor subunit 2, IFNG receptor beta subunit, lysophosphatidic acid receptor 3, *S100A7A*, *S100A8*, and secreted phosphoprotein 1 [1,3,29]. IL1B and IFNG also increase the expression of many endometrial genes involved in PG synthesis and transport and immunity, respectively [1]. Indeed, treatment of endometrial explant tissues with IL1B and IFNG upregulated the expression of SAA3. However, estrogen did not have any effect on the expression of *SAA3* in endometrial explants, suggesting that conceptus estrogen is not involved in the expression of *SAA3* in the endometrium. Overall, these results suggest that conceptus-derived IL1B and IFNG signals could be critical factors for the induction of endometrial *SAA3* expression at the time of conceptus implantation.

The results of this study show that the expression of *SAA3* is epithelial-specific in the endometrium, especially in LE cells on day 12 of pregnancy. This coincides with previous reports that *SAA* expression is primarily localized to epithelial cells in various human tissues, including breast, intestine, lung, kidney, and placenta [30], and to endometrial epithelial cells in cows [31]. In addition, we detected *SAA3* expression in conceptus trophectoderm and endoderm cells during early pregnancy and chorioallantoic tissues during mid to late pregnancy, with increasing level at term. SAAs are an archetypal component of the acute phase response to infection, where they are involved in opsonizing and eliminating invading pathogens and recruiting immune cells such as macrophages and neutrophils to the site of infection by triggering the release of pro-inflammatory cytokines [10]. In mice and humans, SAAs synthesized from intestinal epithelial cells act as opsonins that activate phagocytes by binding to outer membrane protein A of bacteria, and they also show antimicrobial activity [7]. Our previous studies have shown that the maternal endometrium and conceptus tissues during pregnancy in pigs produce a variety of antimicrobial peptides (AMPs), including cathelicidins [32], *S100A7A* [29], *S100A8*, *S100A9*, *S100A12* [33], β-defensin family, peptidase inhibitor 3, and secretory leukocyte protease inhibitor (Lee and Ka, unpublished data). Therefore, our results in this study suggest that SAA3 expressed at the maternal–conceptus interface acts as a component of endometrial and choriallantoic AMPs that protect implantation sites from possible contamination by microorganisms and maintain fertility during pregnancy.

The SCNT technique is a valuable tool for producing genetically valuable or transgenic animals for basic and biomedical use in animal biotechnology, but the high rates of embryonic mortality and pregnancy failure lead to low efficiency in generating viable cloned animals [14,15]. Although the exact cause of low efficiency in SCNT cloning is being investigated, it is thought to be associated with abnormal embryonic reprogramming and inappropriate maternal–conceptus interactions at the maternal endometrium [14,15]. Our previous studies have shown that in endometria with SCNT-derived conceptuses, many genes are abnormally expressed during early pregnancy [16,17,33]. Our results in this study show that *SAA3* expression was significantly lower in endometria with SCNT-derived conceptuses than in those with conceptuses from natural mating on day 12 of pregnancy. Because *SAA3* expression was upregulated by IL1B, which is secreted by the implanting porcine conceptus on day 12 of pregnancy, the decreased endometrial expression of *SAA3* is likely caused by abnormal IL1B secretion by SCNT-derived conceptuses. That dysregulated *SAA3* expression could be involved in increased embryonic death in the endometrium among SCNT-derived conceptuses because SAA3 has important antimicrobial functions, such as immune cell recruitment and regulation of the inflammatory response. Overall, the interactions between the conceptus and the maternal endometrium that are critical for the establishment and maintenance of pregnancy do not occur appropriately in pigs with SCNT-derived conceptuses, and the expression of *SAA3* could be a good biomarker for appropriate implantation and maternal–conceptus interactions during early pregnancy in pigs.

In addition to opsonization and antimicrobial activity, SAA3 induces chemotactic activity, facilitates cholesterol efflux and inflammasome activation, and induces inflammatory or anti-inflammatory cytokines through a variety of specific receptors, including receptor for advanced glycation end products (AGER), formyl peptide receptor 2, P2X purinergic receptor 7 (P2RX7), scavenger receptor class B type 1, toll-like receptor 2 (TLR2), and TLR4 [5,10]. *AGER*, *P2RX7*, *TLR2*, and *TLR4* are expressed at the maternal–conceptus interface in pigs [33] (Cheon and Ka, unpublished data). Therefore, it is likely that SAA3 has pleiotropic functions via specific receptors expressed in the endometrium during the estrous cycle and in the endometrium and chorioallantoic tissues at the maternal–conceptus interface during pregnancy. Especially, the increased expression of SAA3 in chorioallantoic tissues at term may be related to the preparation of parturition by regulating cytokine production.

In conclusion, this study in pigs demonstrated that *SAA3* is expressed at the maternal–conceptus interface in a cell type– and pregnancy stage–specific manner and that the expression of endometrial *SAA3* is induced by conceptus-derived IL1B and IFNG. In addition, the level of *SAA3* expression is diminished in the endometrium with SCNT-derived conceptuses compared to that with natural-mating conceptuses at the time of implantation. Thus, the expression of *SAA3* could be a unique endometrial epithelial marker of conceptus implantation in pigs. Although the exact functions of SAA3 during pregnancy need further investigation, these results indicate that the endometrial expression of *SAA3*, which occurs in response to conceptus signals, could play important roles in regulating endometrial epithelial and placental functions and innate immune responses required for the establishment and maintenance of pregnancy in pigs.

## Figures and Tables

**Table 1:** Summary of primer sequences for real-time RT-PCR, RT-PCR, and in situ hybridization and expected product size

Primer	Sequence of forward (F) and reverse (R) primers (5′→3′)	Annealing temperature (°C)	Product size (bp)	GenBank accession no.
*SAA3*	F: ATCATTTTCTGCTTCCTGATCC	60	262	NM_001044552.1
	R: CTCCATGCTTAAACAAGTCTGTG			
*RPL7*	F: AAG CCA AGC ACT ATC ACA AGG AAT ACA	60	172	NM_001113217
	R: TGC AAC ACC TTT CTG ACC TTT GG			
*UBB*	F: GCA TTG TTG GCG GTT TCG	60	65	NM_001105309.1
	R: AGA CGC TGT GAA GCC AAT CA			
*TBP*	F: AAC AGT TCA GTA GTT ATG AGC CAG A	60	153	DQ845178.1
	R: AGA TGT TCT CAA ACG CTT CG			

RT-PCR, reverse transcription-polymerase chain reaction; *SAA3*, serum amyloid A3; *RPL7*, ribosomal protein L7; *UBB*, ubiquitin B; *TBP*, TATA binding protein.
